# Genetic Structure of Harbour Seals *Phoca vitulina vitulina* Along the Coast of Norwegian Mainland: Implications for Management

**DOI:** 10.1002/ece3.74648

**Published:** 2026-11-07

**Authors:** María Quintela, Anne Kirstine Frie, Roger Lille‐Langøy, Ralph Tiedemann, Kjell Tormod Nilssen

**Affiliations:** ^1^ Institute of Marine Research Group of Population Genetics Bergen Norway; ^2^ Institute of Marine Research Group of Marine Mammals Tromsø Norway; ^3^ Institute of Biochemistry and Biology, Evolutionary Biology & Systematic Zoology University of Potsdam Potsdam Germany

**Keywords:** harbour seals, management, marine mammals, microsatellites, pinnipeds, population structure, stock identification

## Abstract

Alignment between biologically meaningful units and stock boundaries is one or the requirements for sustainable resource management. In Norway, harbour seals ( *Phoca vitulina vitulina* ) are managed at the county level and, when local populations are deemed sufficiently large to support harvesting, hunting is permitted under a quota corresponding to 5% of the counted individuals. An assessment of population structure along the entire Norwegian coastline based upon 16 microsatellites revealed significant overall differentiation (*F*_ST_ = 0.036, *p* < 0.001) consistent with a pattern of Isolation by Distance, in agreement with the estimated effective migration surface (EEMS). Pairwise differentiation among sampling sites challenged the current stock outline. Specifically, (a) geographically large counties (e.g., Finnmark, Nordland) could warrant subdivision into smaller management units, whereas (b) no differentiation was detected across the four south‐eastern counties (Agder, Vestfold, Telemark and Østfold). Positive and statistically significant spatial autocorrelation detected at distances of 0–300 km invoked larger genetical similarity than expected by chance and agreed with the fine‐grained population structure revealed by former tagging studies. The absence of evidence for recent bottleneck events suggests that populations remain at mutation‐drift equilibrium.

## Graphical abstract

A nationwide genetic analysis of harbour seals ( *Phoca vitulina* ) in Norway revealed significant population structure consistent with isolation by distance. The results suggest that some large northern counties may contain multiple distinct management units, while several south‐eastern counties show no detectable differentiation. Overall, the findings indicate that current stock boundaries may not fully reflect biological population structure, although populations appear to remain at equilibrium without signs of recent bottlenecks.

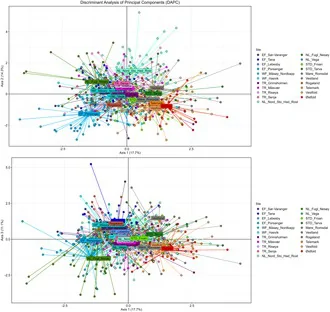

## 1 Introduction

The harbour seal ( *Phoca vitulina* ) is the most ecologically successful pinniped species, owing to several key factors: Its ability to inhabit a broad range of climatic zones, from temperate to Arctic regions (30°–78.5° N in the Atlantic and 28°–61.2° N in the Pacific); its adaptability to diverse aquatic habitats; and its capacity to haul out on different surfaces, including beaches, rocky coasts and sea ice. The taxonomic division into subspecies remains debated (Berta and Churchill 2012; Liu et al. 2022; Sutherland et al. 2024; Westlake and O'Corry‐Crowe 2002). Coastal marine subspecies include *P. v. stejnegeri* (Allen 1902) in the western Pacific Ocean, *P. v. richardii* (Grey 1864) in the eastern Pacific Ocean, and *P. v. vitulina* (Linnaeus 1758) in the Atlantic Ocean. Within freshwater systems, harbour seals inhabiting Iliamna Lake (Alaska) followed a separate evolutionary trajectory resulting in substantial differences from any other marine population (Ferrer et al. 2024), whereas the Ungava harbour seal (*P. v. mellonae*, Doutt 1942) is a recognised subspecies endemic to a freshwater system in Canada (DFO 2018). The current climate changes pose a number of climate‐related challenges on this species, and the fact that harbour seal occupies habitats in close proximity to human populations makes it prone to anthropogenic pressure (see Blanchet et al. (2021) for a comprehensive revision). Despite repeated population declines, harbour seals have responded well to effective conservation measures and thus managed to recolonize former habitats and recover (see Liu et al. 2022 and references therein).

The Atlantic harbour seal (hereafter called harbour seal for simplicity) is a mid‐sized seal with sexually mature males weighing 55–130 kg, and females 45–106 kg, respectively (Bonner 1990). As any other pinniped, harbour seals forage in the aquatic realm whereas breeding, resting and moulting take place in terrestrial habitats including sea ice (Bartholomew 1970). Sexual maturation occurs at around 2–5 years of age for females, and 3–7 years for males (Bigg 1969; Bjørge 1992; Härkönen and Heide‐Jørgensen 1990). Harbour seals aggregate on islands, reefs and sandbanks during breeding in June, and moulting in August. Pups are born on land and—as they shed the white coat (lanugo) in utero—the juvenile pelage allows them to enter the water within a few hours after birth (King 1964). However, they spend most of the time on land the first days and stay with their mothers for a few weeks until they can survive on their own. Ovulation occurs in late lactation, some 3–4 weeks after parturition, and then comes a period of 2–3 months of embryonic diapause followed by about 8 months of active gestation (Reijnders 1990). Harbour seals are opportunistic feeders, foraging in shallow waters close to land (Lowry et al. 2001) and preying on small fish species and young age‐classes of larger fish mainly between 10 and 30 cm in length. In Norwegian waters, the harbour seal diet usually contains 20–25 fish species with variations in the species' composition between areas (Berg et al. 2002; Olsen and Bjørge 1995; Ramasco et al. 2017; Salinger 2021; Sørlie et al. 2020). Their movements also vary, and tagging studies have recorded travel distances ranging from 10 to 250 km (Carroll et al. 2020; Dietz et al. 2013; Florko et al. 2024; Small et al. 2005; Thompson et al. 1996; Tollit et al. 1998; van Neer et al. 2023). For a species capable of long‐distance foraging migrations, movements become more restricted in range and duration during the mating season and they usually return to the same haul‐out region, which reflects in finer population structure than anticipated using genetic markers (see Olsen et al. 2014 and references therein).

This potential for fine‐scale population structure has implications for conservation. Genetic tools can guide efforts towards maintaining—and, where possible, increasing—genetic diversity, a key factor for the long‐term species persistence, particularly in the face of climate change and other anthropogenic pressure. Standing genetic variation can facilitate rapid adaptation (Barrett and Schluter 2008) and may provide harbour seals with the evolutionary potential to cope with environmental perturbations (Andersen and Olsen 2010) or disease outbreaks (Rijks et al. 2008). This potential may be especially important at the edges of species' range where environmental conditions are often suboptimal for growth and reproduction, and directional selection may play a more prominent role in shaping genetic variation (Andersen et al. 2011; Blanchet et al. 2021; Warren et al. 2001). Identifying discrete units of evolutionary or ecological importance and determining whether they require specific management strategies, is therefore particularly relevant (Frankham 2003; Frankham et al. 2002; Moritz 1994, 2002).

However, the delineation of management units relying uniquely on genetic tools has been challenged (Lowe and Allendorf 2010; Palsbøll et al. 2007, 2010; Waples 1998; Waples and Gaggiotti 2006) under the rationale that these methods do not inform about contemporary processes of relevance to management (Palsbøll et al. 2013; Pearse and Crandall 2004). Instead, the use of demographically independent units where population dynamics are determined by local birth and mortality rates rather than immigration from neighbouring populations has been advocated. This issue is directly relevant to mainland Norway, where approximately 7000 harbour seals are distributed with various densities along the entire coast from the Swedish border in the southeast to the Russian border in the northeast. A quota hunt, targeting approximately 5% of the counted numbers, is allowed in areas where the number of individuals is deemed large enough to sustain hunting. Harbour seals are also caught incidentally in fish gillnets and lobster traps and, although almost no by‐catches are reported, 555 harbour seals have been estimated to be caught annually in Norwegian fish gillnets (Bjørge et al. 2017).

Harbour seal management units in Norway are delineated by county borders based upon a combination of administrative criteria and dispersal‐distance data derived from mark‐recapture experiments (Bjørge et al. 2002). However, Norwegian counties differ considerably in coastline length as well as in numbers and size of islands and fjords. The coastline also spans a pronounced environmental gradient, from the waters of the southern Skagerrak to the fjord and open‐coast habitats of northern Norway. These geographic and environmental differences may therefore encompass multiple genetically distinct populations, raising questions about whether the current management regime should be revised. To address this, the present study investigates genetic population structure in Norwegian harbour seals and aims to provide guidance for delineating biologically meaningful management units.

## 2 Materials and Methods

### 2.1 Sampling

Newborn harbour seal pups were caught in the sea as well as on reefs and sandbanks and sampled using a biopsy puncher to cut a small piece (ca. 3–5 mm) from the edge of the thin tissue between the claws at one of the hind flippers. On land, the sample was taken by one person using one hand holding one hind flipper and using the biopsy puncher with the other hand. In the water, two persons (one boat‐driver) in an inflatable boat (Zodiac) caught the pup using a landing net operated by the person in front. Sampling was carried out during good weather periods with no wind and rain. Pups were sampled during the pupping/breeding period in June 2007 and 2011–2025 at the main breeding sites ranging from Finnmark County in the north along the entire coast south to the outer Oslofjord. In addition, tissue samples from adult seals were obtained from animals harvested in the traditional hunt in Finnmark County. Tissue samples were stored in EtOH 96% prior to DNA isolation, and geographic coordinates were recorded for each individual.

### 2.2 DNA Isolation and Genotyping

DNA was extracted using the Qiagen DNeasy 96 Blood & Tissue Kit in 96‐well plates, each of which contained two or more negative controls, and concentration was quantified using a NanoDrop 8000. A panel of 17 microsatellites developed both for *Phoca vitulina* as well as for other pinniped species was multiplexed in four reactions (Table S1). PCR amplifications were performed in a final volume of 5 μL containing approximately 20 ng DNA template, 1× buffer, 2 mM MgCl_2_, 0.2 mM dNTPs, 0.2–0.5 μM of each primer and 1U GoTaq polymerase in an Applied Biosystems Gene Amp PCR Systems 2700 thermal cycler. PCR profiles consisted of 5‐ or 15‐min denaturation at 95°C, followed by 30–32 cycles of 30 s denaturation at 95°C, 60 s at 55°C or 58°C annealing temperature, and 60 s extension at 72°C, with a final step of 15 min at 72°C or 30 min at 60°C for multiplexes 1 (PvPP1) and 4 (PvTP). In multiplexes PvPP1 and PvTP, QIAGEN Multiplex PCR Kit (#206143) was used. PCR products were analysed on an ABI 3730 Genetic Analyser and the 500LIZTM size standard (Applied Biosystems) was used to accurately determine the size of the fragments and allelic variation. Scoring was performed using the software GeneMapper Software 6 (ThermoFisher Scientific). Automatically binned alleles were manually checked by two researchers prior to exporting data for statistical analyses.

Reproducibility was tested on six individuals at all markers as well as on 96 individuals at two of the multiplex reactions containing a total of ten loci. Locus SGPV3 was discarded, as in Steinmetz et al. (2023), due to genotyping inconsistencies during cross‐laboratory validation. Otherwise, no mismatches were detected. The threshold of acceptance of missing data was set at 30% to retain as many individuals as possible per sample. After discarding 23 individuals due to poor amplification, the final dataset consisted of 573 seals, 78% of which displayed no missing data. Individuals were distributed into pups (*N* = 369) and adults (*N* = 204). Statistical analyses were performed combining individuals of both age‐classes. In five of the samples, pups were absent/virtually absent whereas the percentage of pups ranged between 84% and 100% in 61% of the samples (see Table 1).

**TABLE 1:** Sample summary statistics obtained for the total set of individuals genotyped at 16 microsatellite loci: Samples per county, total number of individuals (*N*) and number (and percentage) of pups (*N*_PUPS_), percentage of polymorphic loci, total number of alleles (*N*_A_), ratio between alleles and sampling size (*N*_A_/*N*), allelic richness based on the minimum sample per locus and on 20 diploid individuals, respectively; number of private alleles; observed heterozygosity, *H*_o_ (mean ± SE); unbiased expected heterozygosity, u*H*_e_ (mean ± SE); inbreeding coefficient, *F*_IS_ (mean ± SE); number of deviations from Hardy–Weinberg equilibrium (HWE) at α = 0.05; number of deviations from Linkage Disequilibrium (LD) at α = 0.05 both before and after False Discovery Rate (FDR) correction.

County	Sample	*N*	*N*_PUPS_ (%)	Polym loci	*N*_A_	*N*_A_/*N*	AR (*N* = min)	AR (*N* = 20)	Priv alleles	*H*_o_	u*H*_E_	*F*_IS_	HWE	LD	*p H*_exc_
Finnmark	EF_Sør‐Varanger	25	0 (0)	100	55	2.2	2.6	3.3	0	0.468 ± 0.045	0.485 ± 0.039	0.016 ± 0.041	4 (0)	6 (0)	0.570
Finnmark	EF_Tana	25	20 (80)	100	51	2.0	2.4	3.2	1	0.452 ± 0.047	0.472 ± 0.031	0.042 ± 0.065	2 (1)	3 (0)	0.510
Finnmark	EF_Lebesby	14	0 (0)	100	47	3.4	2.4	na	1	0.479 ± 0.050	0.488 ± 0.039	−0.038 ± 0.073	2 (0)	5 (0)	0.161
Finnmark	EF_Porsanger	51	1 (2)	100	56	1.1	2.4	3.1	0	0.433 ± 0.052	0.440 ± 0.043	0.029 ± 0.041	2 (1)	13 (0)	0.826
Finnmark	WF_Måsøy_Nordkapp	14	0 (0)	100	50	3.6	2.5	na	0	0.427 ± 0.054	0.457 ± 0.049	0.041 ± 0.078	1 (1)	1 (0)	0.702
Finnmark	WF_Hasvik	29	15 (51.7)	100	55	1.9	2.6	3.3	0	0.486 ± 0.048	0.493 ± 0.044	−0.009 ± 0.040	1 (0)	4 (0)	0.232
Troms	TR_Grimsholmen	15	15 (100)	93.75	50	3.3	2.5	na	0	0.458 ± 0.045	0.504 ± 0.047	0.048 ± 0.038	0 (0)	3 (0)	0.151
Troms	TR_Måsvær	26	26 (100)	100	62	2.4	2.6	3.7	1	0.499 ± 0.044	0.477 ± 0.041	−0.072 ± 0.032	0 (0)	7 (0)	0.912
Troms	TR_Risøya	21	21 (100)	100	54	2.6	2.6	3.4	2	0.522 ± 0.044	0.507 ± 0.042	−0.059 ± 0.043	0 (0)	6 (0)	0.264
Troms	TR_Senja	24	22 (91.7)	100	53	2.2	2.6	3.3	0	0.487 ± 0.029	0.510 ± 0.032	−0.004 ± 0.052	1 (1)	6 (0)	0.248
Nordland	NL_Nord_Sto_Had_Rost	58	56 (96.6)	100	67	1.2	2.7	3.5	3	0.521 ± 0.027	0.519 ± 0.025	−0.015 ± 0.024	0 (0)	16 (1)	0.894
Nordland	NL_Fugl_Nesøy	41	39 (95.1)	100	61	1.5	2.6	3.5	1	0.446 ± 0.040	0.481 ± 0.041	0.060 ± 0.034	4 (1)	3 (0)	0.702
Nordland	NL_Vega	18	17 (94.4)	100	54	3.0	2.6	na	2	0.461 ± 0.043	0.466 ± 0.042	−0.023 ± 0.040	1 (0)	5 (0)	0.839
Trøndelag	NTD_Hortavaer	6	6 (100)	87.5	38	6.3	2.3	na	0	0.310 ± 0.056	0.384 ± 0.063	0.054 ± 0.086	1 (0)	0 (0)	0.879
Trøndelag	STD_Froan	23	23 (100)	100	51	2.2	2.5	3.2	0	0.496 ± 0.045	0.495 ± 0.043	−0.034 ± 0.047	0 (0)	8 (0)	0.116
Trøndelag	STD_Tarva	20	19 (95.0)	100	50	2.5	2.4	2.8	0	0.456 ± 0.054	0.460 ± 0.046	0.001 ± 0.050	2 (0)	0 (0)	0.391
Møre_Romsdal	Møre_Romsdal	37	31 (83.8)	100	59	1.6	2.7	3.4	1	0.501 ± 0.040	0.523 ± 0.040	0.020 ± 0.036	1 (1)	3 (1)	0.248
Vestland	Vestland	19	18 (94.7)	100	47	2.5	2.4	na	0	0.459 ± 0.044	0.462 ± 0.028	0.000 ± 0.055	0 (0)	7 (0)	0.149
Rogaland	Rogaland	21	19 (90.5)	93.75	46	2.2	2.3	2.9	0	0.453 ± 0.064	0.441 ± 0.049	−0.028 ± 0.046	1 (0)	5 (1)	0.211
Agder	Agder	5	0 (0)	93.75	43	8.6	2.6	na	0	0.488 ± 0.066	0.469 ± 0.056	−0.158 ± 0.070	0 (0)	0 (0)	0.805
Telemark	Telemark	24	10 (41.7)	100	52	2.2	2.5	3.2	1	0.465 ± 0.044	0.472 ± 0.039	−0.005 ± 0.044	0 (0)	7 (0)	0.316
Vestfold	Vestfold	34	9 (26.5)	100	57	1.7	2.5	3.3	2	0.460 ± 0.043	0.474 ± 0.036	0.020 ± 0.043	0 (0)	5 (0)	0.666
Østfold	Østfold	23	10 (43.5)	100	54	2.3	2.6	3.3	0	0.455 ± 0.044	0.479 ± 0.044	0.018 ± 0.035	0 (0)	6 (0)	0.510

*Note:* Bottleneck results: Probability of heterozygote excess (*p H*_exc_) calculated using Wilcoxon tests under a two‐phase mutation model (TPM) conducted with 90% single‐step mutations and 10% multi‐step mutations. Samples shaded in grey have *N* ≤ 6.

### 2.3 Statistical Analysis

Individuals were arranged into samples following the coastline (see Figure 1 as well as Figure S1a–f for more detailed information about sampling locations). As the aim of this study was to test if the current arbitrary management units based upon political divisions aligned with the biological units, individuals were distributed into samples following the eleven coastal counties in which Norway is divided, i.e., from North to South: Finnmark (F), Troms (TR), Nordland (NL), Trøndelag (T), Møre‐Romsdal, Vestland, Rogaland, Agder, Telemark, Vestfold and Østfold. Vast counties were subdivided into East and West in the case of Finnmark (EF_ and WF_), or North and South in Trøndelag (NTD_ and STD_). In total, 23 samples with sizes ranging between *N* = 5 and *N* = 58 (see Table 1) were obtained. The two samples with *N* ≤ 6 (NTD_Hortavaer and Agder) were dismissed from part of the analyses due to their limited statistical power.

**FIGURE 1:**
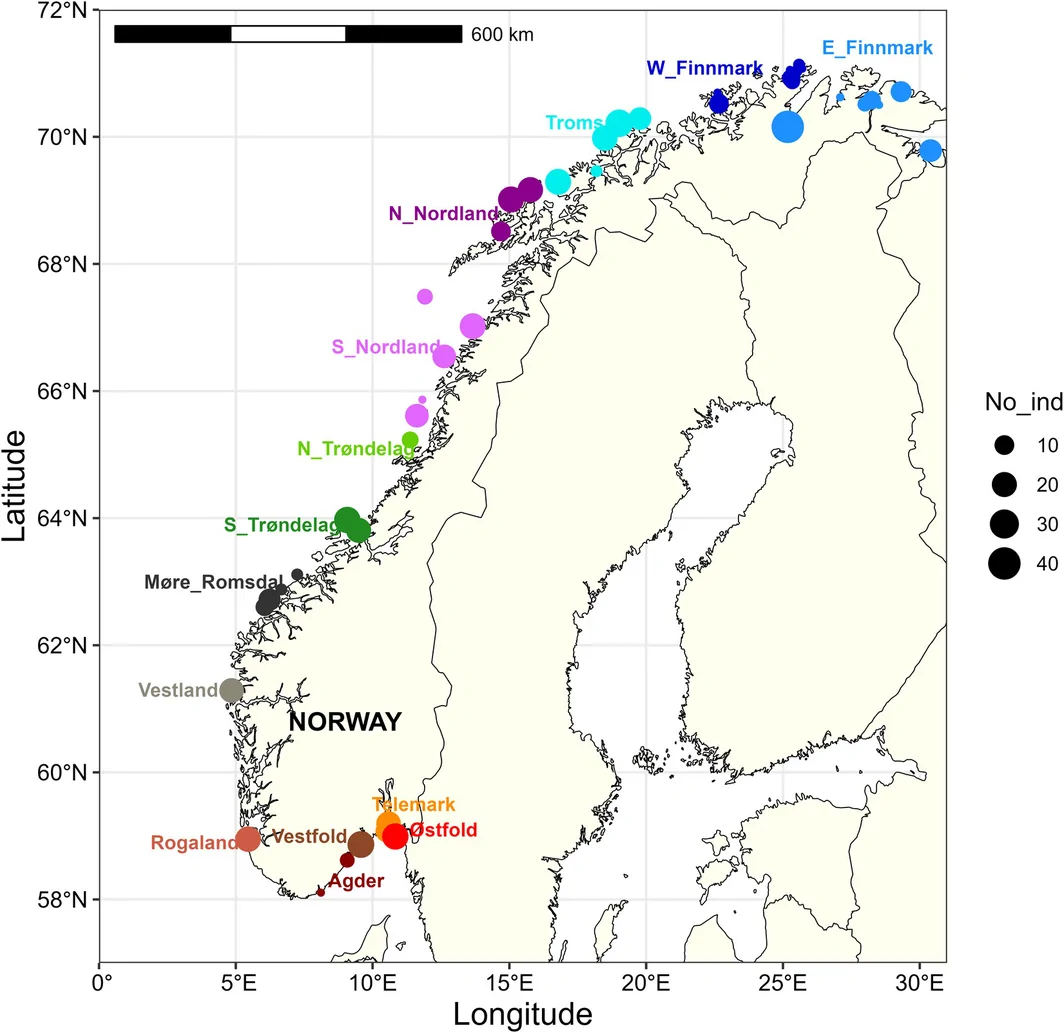
Map of sampling locations of harbour seal *Phoca vitulina vitulina* along the Norwegian coast created with the *geom_sf()* function implemented in the R package *ggplot2* (Wickham 2016). Detailed maps of the different counties can be found in the Figure S1a–f.

To assess if the panel of 16 microsatellites would accurately discriminate between individuals in a population, the genotype accumulation curve was built using the function *genotype curve* in the R (R Core Team 2025) package *poppr* (Kamvar et al. 2014) by randomly sampling loci 1000 times without replacement and counting the number of observed multilocus genotypes (MLGs).

Loci carrying signatures of locally divergent selection not only provide insight into evolutionary processes but might function as powerful markers to evaluate spatially explicit genetic structure for management outline (Nielsen et al. 2009; Russello et al. 2012). Loci departing from neutrality were identified using a combination of BayeScan 2.1 (Foll 2012; Foll and Gaggiotti 2008) and Arlequin v.3.5.1.2 (Excoffier et al. 2005). Analyses on Arlequin were conducted based on 100 demes with 50,000 simulations under a hierarchical island model. In BayeScan, sample size was set to 10,000, burn in was set at 50,000, the thinning interval to 50 resulting in 559,000 total interactions. Loci with log_10_(BF) > 2 (i.e., ‘decisive selection’ (Foll and Gaggiotti 2006)) were retained as outliers.

The total number of alleles, number of private alleles, and allelic richness per sample was calculated using MSA 4.05 (Dieringer and Schlötterer 2003). The inbreeding coefficient (*F*_IS_), observed (*H*_O_) and unbiased expected heterozygosity (*uH*_E_), i.e., expected proportion of heterozygotes adjusted to remove the bias introduced by sampling in samples with low N, were computed for each sample with GenAlEx v6.1 (Peakall and Smouse 2006). The genotype frequency of each locus and its direction (heterozygote deficit or excess) was compared with Hardy–Weinberg expectations (HWE) using the program GENEPOP 4.0.6 (Rousset 2008) as was linkage disequilibrium (LD) between pairs of loci. The False Discovery Rate (FDR) correction of Benjamini and Hochberg (1995) was applied to *p*‐values to control for Type I errors.

Pairwise *F*_ST_ (Weir and Cockerham 1984) was computed using ARLEQUIN (Excoffier and Lischer 2010) and visualised through a heatmap coupled with a dendrogram, with their corresponding *p*‐values calculated after 10,000 permutations. The relationship among geographically explicit samples was examined using the Discriminant Analysis of Principal Components (DAPC) (Jombart et al. 2010) implemented in the R (R Core Team 2025) package *adegenet* (Jombart 2008). To avoid overfitting, both the optimal number of principal components and discriminant functions to be retained were determined using the cross validation function (Jombart and Collins 2015; Miller et al. 2020). Furthermore, the Bayesian clustering approach implemented in STRUCTURE v.2.3.4 (Pritchard et al. 2000), and conducted using the software ParallelStructure (Besnier and Glover 2013), was used to identify genetic groups under a model assuming admixture and correlated allele frequencies with and without using LOCPRIORS to assist the clustering. Ten runs with a burn‐in period consisting of 100,000 replications and a run length of 1000,000 MCMC iterations were performed for *K* = 1 to *K* = 10 clusters. To determine the number of genetic groups, STRUCTURE output was analysed using two approaches: (a) the *ad hoc* summary statistic ΔK of Evanno et al. (2005), and (b) the Puechmaille (2016)'s four statistics (MedMedK, MedMeanK, MaxMedK and MaxMeanK), both implemented in StructureSelector (Li and Liu 2018). Finally, the ten runs for the selected Ks were averaged with CLUMPP v.1.1.1 (Jakobsson and Rosenberg 2007) using the FullSearch algorithm and the G' pairwise matrix similarity statistic, and graphically displayed using barplots.

Genetic structure was also assessed with Population Graphs, a graph‐theoretic approach that uses conditional genetic distance (Dyer and Nason 2004) as the response variable, which is primarily created by both gene‐flow and shared ancestry. Population Graphs has been shown to capture underlying demographic processes more accurately than methods based on pairwise estimates of genetic structure or genetic distance metrics (Dyer et al. 2010). The identification of genetic covariance structure among populations is independent of evolutionary assumptions aiming to minimise Hardy–Weinberg and linkage disequilibrium within populations (Dyer and Nason 2004). Population Graphs were constructed using the R packages *popgraph* and *gstudio* (Dyer 2015a, 2015b) and topological analyses were performed using the *igraph* package (Dyer 2015c). Graph distance was estimated as the minimal topological distance connecting nodes (i.e., pairs of sampling sites). The distances between nodes in the network, based on genetic covariance, were evaluated in relation to their physical separation measured as the shortest waterway distance under a model of isolation by graph distance, IBGD (Dyer and Nason 2004). A relatively small graph distance between spatially distant sites indicates long‐distance gene‐flow (extended edges). Conversely, geographical or ecological barriers that impede gene‐flow relative to other localities with similar distances generate relatively high graph distances (compressed edges). Correlations and detection of extended and compressed edges were determined by regressing geographic and graph distances. Analyses were restrained to samples with *N* > 6.

The relationship between geographic and genetic distance, *F*_ST_/(1−*F*_ST_) was examined to investigate if it followed the expectations of an ‘Isolation by Distance’ pattern (IBD), i.e., increasing genetic differentiation with geographic distance as a result of restricted gene flow and drift (Rousset 1997; Slatkin 1993; Wright 1943). A two‐tailed Mantel (1967) test was conducted using PASSaGE v2 (Rosenberg and Anderson 2011) and significance was assessed via 10,000 permutations. The matrix of pairwise shortest geographic distance by water (i.e., avoiding land masses) was calculated with the R (R Core Team 2025) package *marmap* (Pante and Simon‐Bouhet 2013).

Patterns consistent with Isolation‐by‐Distance (IBD) might hide local deviations that reflect either barriers to gene flow or zones of increased contact (corridors). EEMS, Estimated Effective Migration Surfaces (Petkova et al. 2016) provides a way to investigate these deviations. It uses individual‐based migration rates to estimate and visualise regions where effective migration is higher or lower than expected under the overall IBD pattern, highlighting areas where gene flow may be facilitated or impeded. EEMS surfaces were constructed using the script for microsatellites analysis *runeems_sats* available from Github (https://github.com/dipetkov/eems). Using more demes creates a denser grid and a more flexible EEMS model with more migration‐rate parameters (Petkova 2017). Thus, after testing for MCMC convergence using different parameter combinations, the following conditions were selected: 5,000,000 burn in steps followed by 10,000,000 MCMC iterations sampled every 10,000 steps. Three runs with 50, 100, 200, 300 and 400 demes each were conducted using different seeds and averaged to produce the final EEMS surface using the R (R Core Team 2025) *rEEMSplots* package.

Spatial structure of genetic variation across intervals of 100 km of distance classes generated by *marmap* (Pante and Simon‐Bouhet 2013) was explored using GenAlEx v6.1 (Peakall and Smouse 2006). The autocorrelation coefficient (*r*) ranges from 0 to 1, with 0 indicating that autocorrelation does not differ from random expectations. Intervals at 95% confidence were generated using 999 permutations.

GeneClass 2.0 (Piry et al. 2004) was used both to calculate the percentage of individuals correctly self‐assigned to their sample of origin as well as to conduct direct assignment of the individuals to their county of provenance.

As harbour seals are known to experience extreme fluctuations driven by epidemic outbreaks, bottleneck events were investigated using BOTTLENECK v1.2 (Piry et al. 1999). When populations decline in numbers, the reduction in the number of alleles happens faster than the loss of heterozygosity, leading to a seemingly heterozygosity excess (Cornuet and Luikart 1996). Analyses were conducted under a two‐phase mutation model (TPM) (Di Rienzo et al. 1994), the most appropriate for microsatellite loci as it assumes that even if most of the new alleles evolve from existing ones by loss or gain of a single repeat unit (one‐step change: Stepwise Mutation Model, SMM), a small proportion evolve through multi‐step changes (like Infinite Alleles Model, IAM), therefore making it more realistic than any of the models alone. Sensitivity analyses were conducted using different proportions of single‐step mutations (70%, 80%, 85%, 90%, 95%) under two different variances (12% and 36%). For comparative purposes, the parameters used by Andersen et al. (2011) in a microsatellites study of harbour seals in the same geographic area were retained; i.e., 90% single‐step mutations, 10% multi‐step mutations and a variance of 12%. The test for bottlenecks was performed using the Wilcoxon test (Piry et al. 1999), which assesses whether the number of loci with heterozygote excess is larger than expected by chance. This non‐parametric test is suited for analyses involving few loci, in contrast with the standard deviation test that requires ≥ 20 loci (Andersen et al. 1998). Model‐shift tests were evaluated in all cases.

## 3 Results

### 3.1 Genetic Diversity and Outlier Detection Analyses

The 16 microsatellites segregated into a total of 94 alleles, ranging from two to nine per locus (Table S1). The genotype accumulation curve reaching a plateau at 8 loci indicated that 50% of the total markers could differentiate unique individuals in a sample (Figure S2). No locus was found to be under positive selection according to Arlequin and, although BayeScan flagged ten loci as putative outliers under balancing selection (Table S2), no consensus was found; therefore, all loci were retained for further analyses.

Allelic richness computed with the minimum number of individuals per locus ranged between 2.3 and 2.7 across all samples (Table 1). However, when considering *N* ≥ 20, the samples of STD_Tarva and Rogaland displayed the lowest allelic richness (2.8 and 2.89 respectively) whereas the largest (3.7) was detected in more northern samples TR_Måsvær followed by NL_Nord_Sto_Had_Rost and NL_Fugl_Nesøy, with 3.5. Only ten samples displayed private alleles, ranging between 1 and 3 (Table 1). Deviations from HWE were detected in 23 of the 364 alleles per sample tests (6.3%) and dropped to 6 (1.6%) after FDR correction. Locus TBPV2 showed the largest deviations from HWE, which affected to 4 out of the 23 total samples. In EF_Sør‐Varanger and NL_Fugl_Nesøy four loci deviated from HWE whereas in the remaining samples the number of deviating markers ranged between 0 and 2 (Table 1). Likewise, 119 out of the 2760 tests for LD (4.3%) showed significant linkage, which dropped to 3 (0.1%) after FDR correction. Both observed and expected heterozygosity as well as inbreeding coefficient took similar values across samples. The negative *F*_IS_ values found in half of the samples were suggestive of outbreeding whereas the largest values ranging between 0.04 and 0.06 could suggest moderate levels of inbreeding.

### 3.2 Population Structure and Assignment

AMOVA restricted to the samples with *N* ≥ 6 revealed significant differentiation (*F*_ST_ = 0.036, *p* < 0.001) with 3.6% of the variation hosted among samples. Most of the *F*_ST_ pairwise comparisons (87%) were significantly different from zero (Table S3). However, almost no genetic differentiation was detected neither in the geographic area between EF_Sør‐Varanger and WF_Måsøy_Nordkapp in the north, nor in the area between Telemark and Østfold in the south. The first dichotomic division of *F*_ST_ dendrogram separated Østfold, Vestfold, Telemark, Vestland and STD_Tarva from all remaining samples (Figure 2). Within the latter, two subgroups could be found, the smallest one containing EF_Porsanger, NL_Vega, Rogaland and WF_Måsøy_Nordkapp. Interestingly, Rogaland clustered with some of the northernmost locations.

**FIGURE 2:**
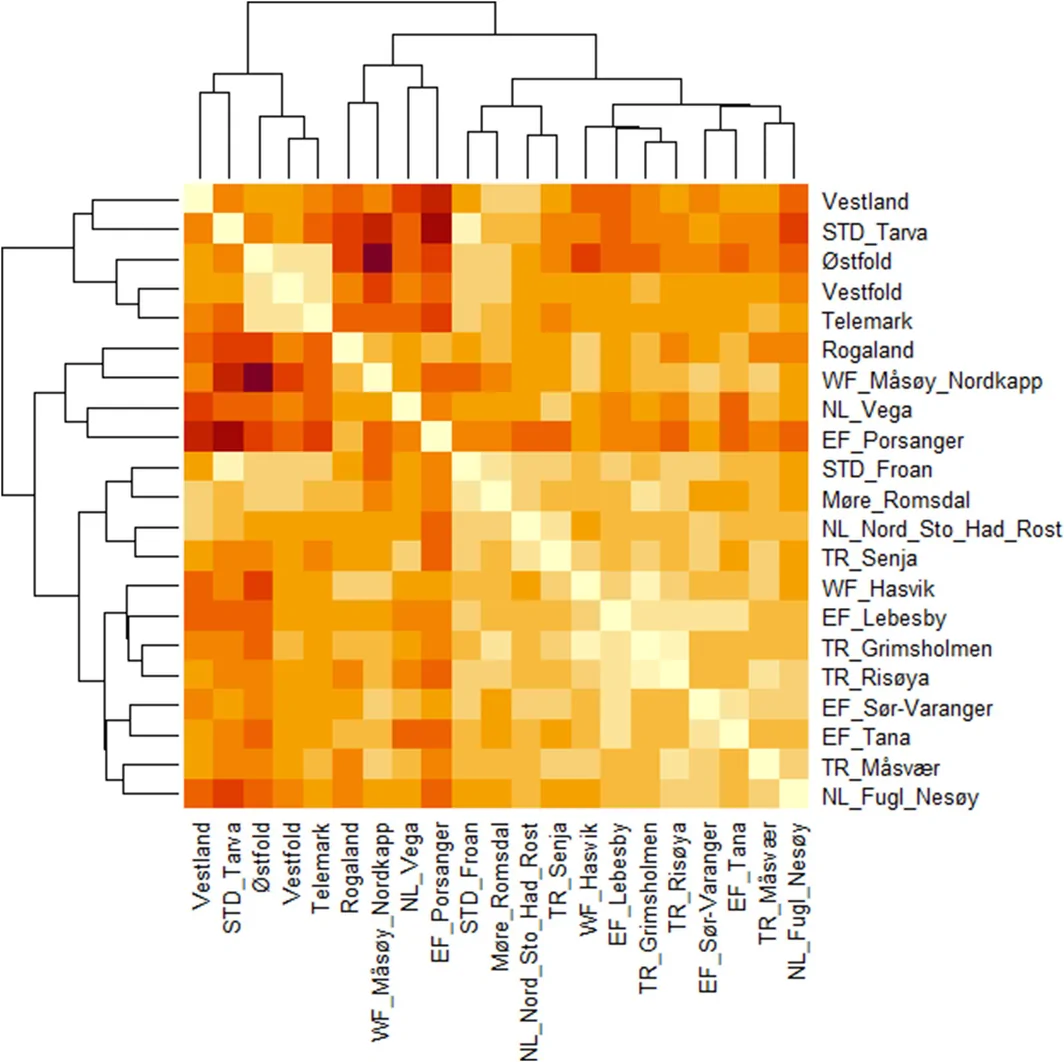
Heatmap coupled with dendrogram of Weir and Cockerham (1984)'s pairwise *F*_ST_ between geographically‐explicit samples of 562 individuals genotyped at 16 microsatellites. The darker the colour, the stronger the differentiation it depicts. *F*_ST_ values and their corresponding probabilities can be found in the Table S3. Samples with *N* ≤ 6 were excluded from this analysis.

The first axis of differentiation of the DAPC, accounting for 17.7% of the variation, distributed samples following a latitudinal gradient, with the exception of Rogaland, which clustered with the northern samples (Figure 3, upper plot). The second axis (14.3%) slightly distinguished the centroid of EF_Porsanger from the other samples, whereas the third axis of variation (11.1.5%) achieved a similar separation for the centroid of NL_Fugl_Nesøy (Figure 3, lower plot).

**FIGURE 3:**
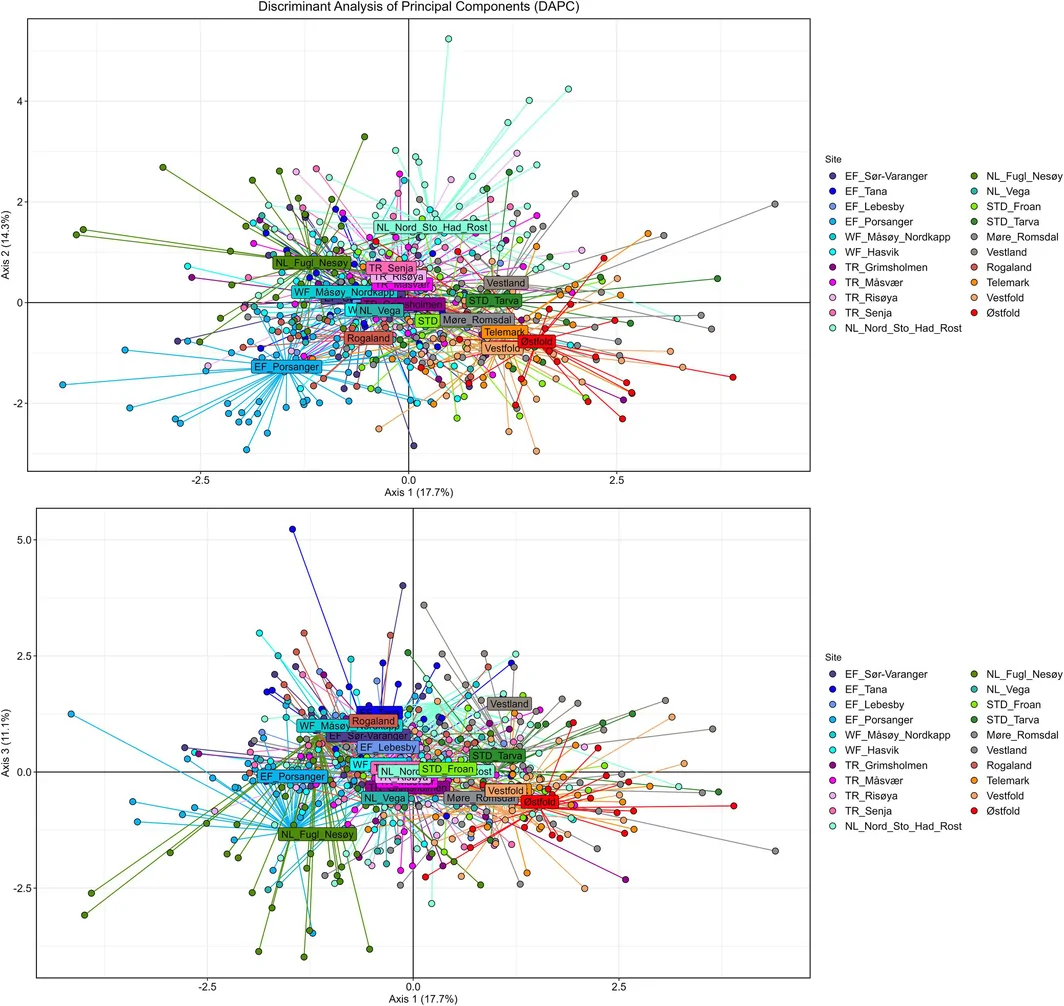
Genetic differentiation among 562 individuals genotyped at 16 microsatellites distributed in geographically explicit samples using Discriminant Analysis of Principal Components (DAPC) after retaining 40 principal components and 3 discriminant functions as described by axes 1 and 2 (upper plot) and axes 1 and 3 (lower plot). Samples with *N* ≤ 6 were excluded from this analysis.

Puechmaille and Evanno's methods conducted upon STRUCTURE outcome provided various solutions including *K* = 2, 3, 5, 6 and 7 (Figure S3). The use of geographic information proved essential to refine STRUCTURE's resolution (see Figure S4 for barplots without priors). Assisted clustering revealed solutions such as *K* = 2 (Evanno's method) or *K* = 6, 7 (Puechmaille's statistics) although differences were marginal in the range *K* = 4–7 (Figure S5). The first level of division at *K* = 2 showed a certain gradient of latitudinal differentiation interrupted by NL_Fugl_Nesøy/NL_Vega and Rogaland, which seemed genetically closer to Finnmark than expected (Figure 4, upper plot). At *K* = 5, in addition to the underlying N‐S gradient, distinct groups emerged such as: EF_Porsanger, NL_Fugl_Nesøy to NTD_Hortavaer, or the geographic area between Agder and Østfold (Figure 4, lower plot). Again, Rogaland showed greater similarities to Finnmark than to the geographically adjacent locations, in agreement with the lower pairwise *F*_ST_ values towards the northern samples, particularly EF_Porsanger (*F*_ST_ = 0.015, *p* = 0.019).

**FIGURE 4:**
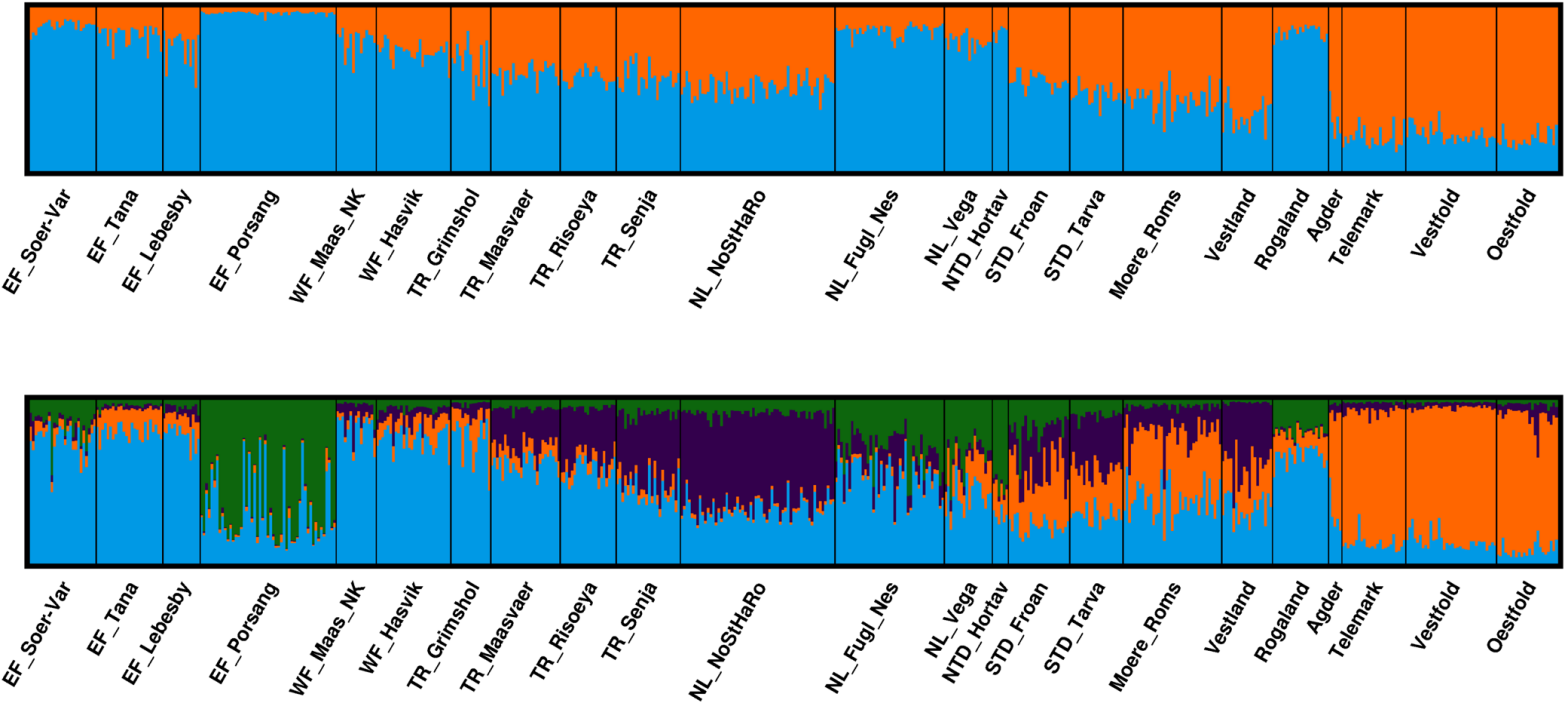
Barplot representing the proportion of individuals' ancestry to cluster of all 573 individuals genotyped at 16 microsatellites at *K* = 2 (upper plot) and *K* = 5 (lower plot) as inferred from STRUCTURE using geographic information to assist the clustering.

The distribution of spatial genetic structure suggested restricted gene‐flow under a model of isolation by graph distance (IBGD; Spearman's rho = 0.218, *p =* 0.0015; Figure S6). Popgraph depicted a very sparse web where the 21 nodes (samples) were connected by 51 edges out of the 210 possible ones (Figure 5). The number of connections per sample ranged from 2 in Telemark to 8 in EF_Porsanger (Table S4) The 51 edges were distributed into 29 compressed (i.e., less gene‐flow than expected given geographic distance), 16 extended (i.e., more gene‐flow than expected given geographic distance) and 6 proportional. Samples from Finnmark area were mostly connected with the northernmost counties such as Troms (6 connections) and Nordland (5 connections). However, 4 extended edges connect Finnmark with the sample from Rogaland, in a geographic range of distances of 1600–1900 km depicting that the gene flow between these sites was larger than expected for such geographic distance.

**FIGURE 5:**
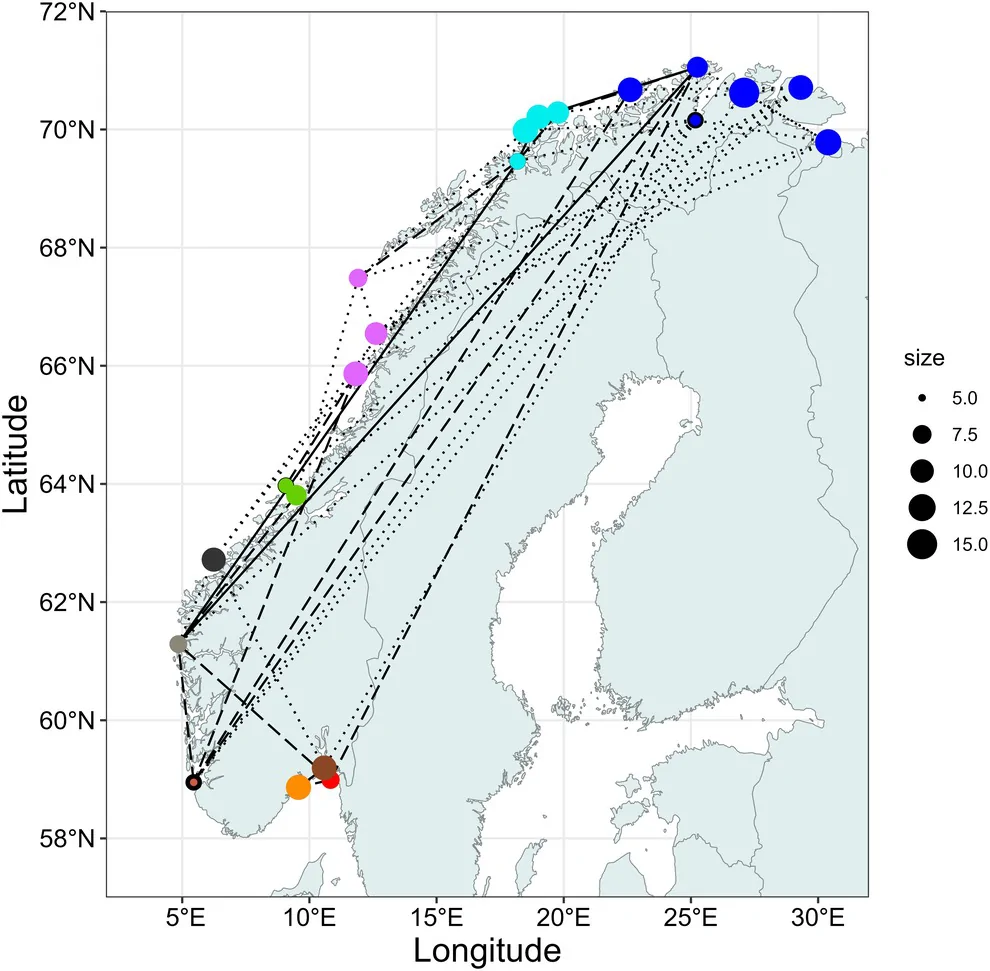
Population Graph representing the distribution of genetic covariance in harbour seal samples based upon 16 microsatellites. Nodes represent samples with different relative sizes depending on the amount of within‐population genetic variance and colours that depict different counties. Edges connecting the nodes represent the pattern of genetic covariance among populations: Dashed lines denote extended edges (i.e., more gene‐flow than what would be expected given geographic distance), dotted lines compressed edges, (i.e., less gene‐flow than what would be expected given geographic distance).

A highly significant correlation (*r* = 0.41, *p* < 0.001) resulting in a pattern of Isolation by Distance (Figure 6) was detected when confronting *F*_ST_/(1−*F*_ST_) and the shortest water distance (km) with 17% of the variation in genetic distance explained by the geographic distance. The two samples with *N* ≤ 6 were excluded from this analysis for lacking statistical power to accurately assess pairwise *F*_ST_. The low genetic differentiation between Rogaland and certain Finnmark samples, despite the large geographical distance separating them, was illustrated with red dots in the scatterplot.

**FIGURE 6:**
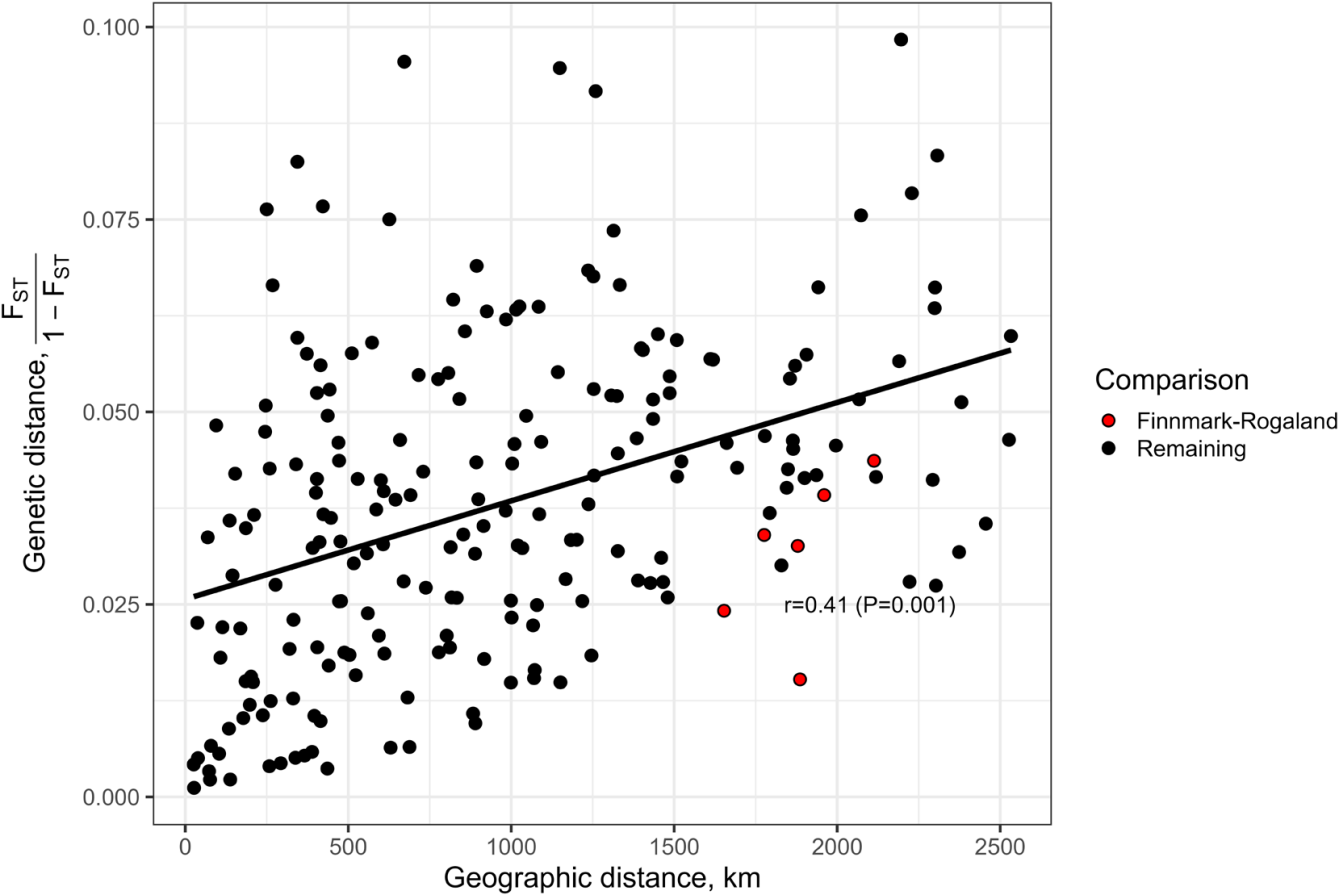
Mantel correlation between pairwise shortest distance by water (km) and pairwise genetic differentiation (*F*_ST_/(1−*F*_ST_)) computed using the set of 16 microsatellite loci and restricted to samples with *N* ≥ 6. Red dots depict the relation between Rogaland and the samples from Finnmark whereas the black dots correspond to the remaining comparisons.

The average EEMS surface across the fifteen converged chains was broadly consistent with an isolation‐by‐distance model, while highlighting areas of modest deviations from this pattern (Figure 7). Although no strong barriers to gene flow or migration corridors were detected (Figure S7), the broad, deep fjord in Porsanger (Finnmark) was signalled as an area of reduced relative migration (Figure 7).

**FIGURE 7:**
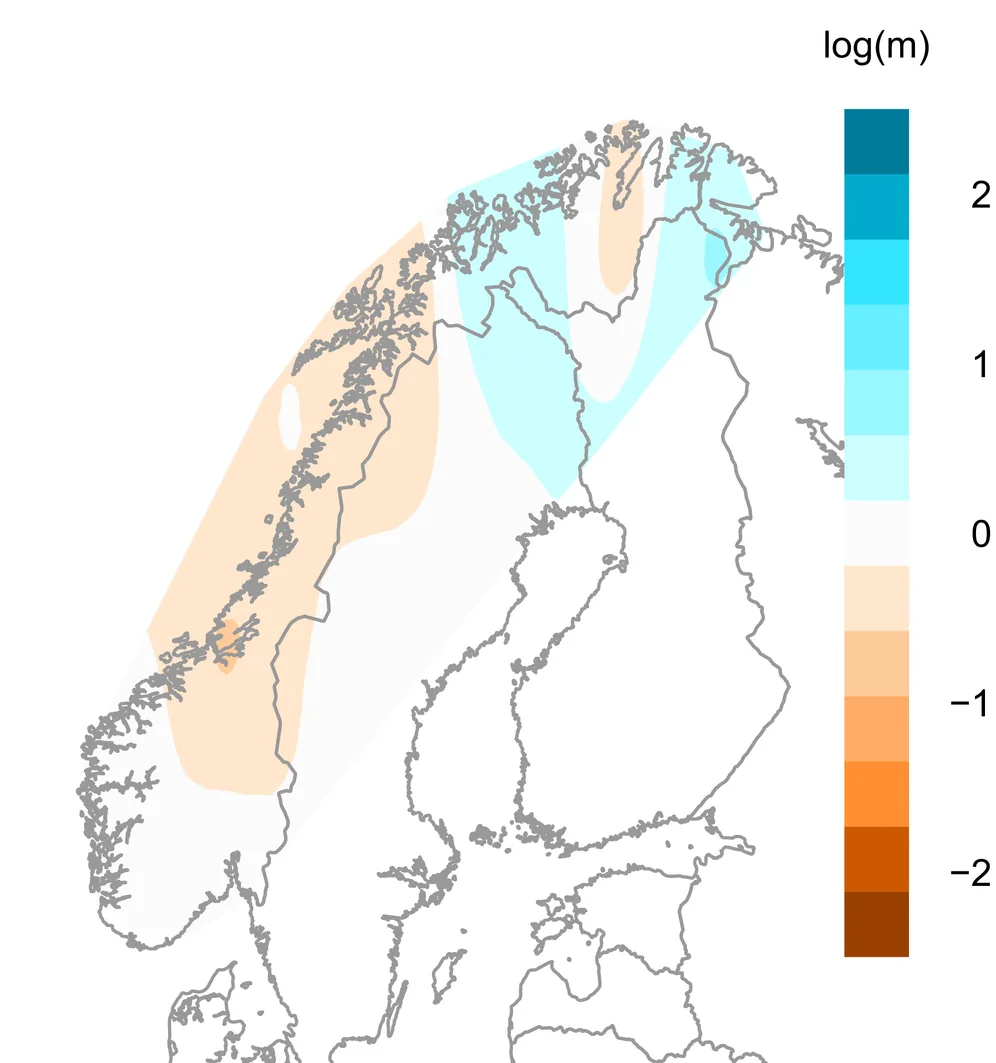
Individual‐based EEMS analysis of effective migration rates (m) represented on a log_10_ scale. Areas showing negative values (orange) correspond to areas of reduced gene‐flow with regards to the expectations of an Isolation‐by‐Distance pattern (white background). Similarly, blue colour depicts areas of increased gene flow. Migration surfaces result from the average of 15 runs conducted with a variable number of demes (from 50 to 400).

Spatial autocorrelation was positive and statistically significant in the range between 0 and 300 km of distance (Figure 8) indicating that individuals within this geographic range were genetically more similar than expected by chance. The neighbourhood size, calculated as the first distance class where the confidence interval crosses zero, was of 300 km. The negative correlation detected in the ranges between 1200 and 2500 km invoked IBD. However, the non‐significant negative correlation observed at distance of approximately 1700–1900 km, together with *r* approaching zero at 1800 km, seemed largely driven by the low genetic differentiation between Rogaland and the Finnmark samples, namely EF_Porsanger.

**FIGURE 8:**
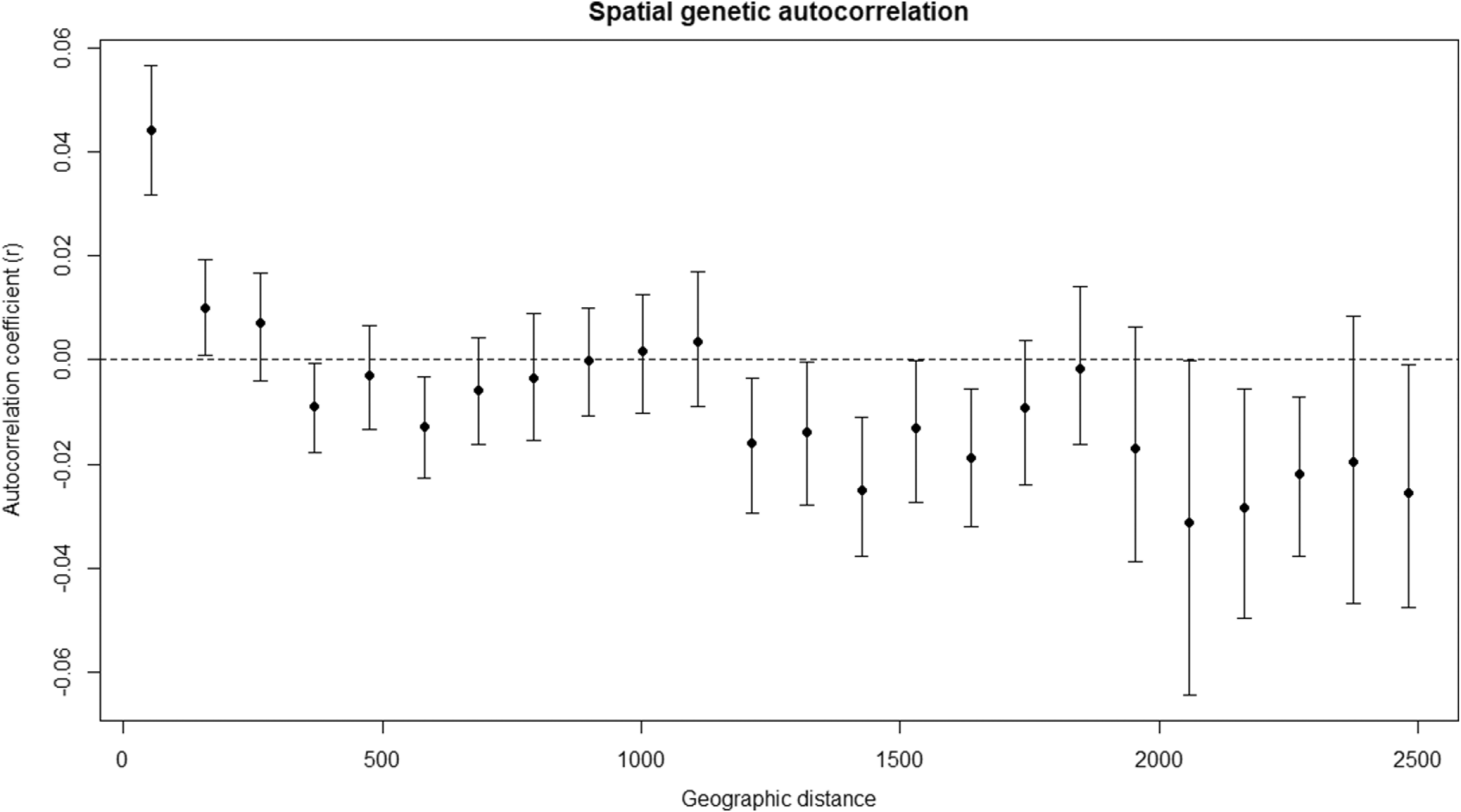
Spatial autocorrelogram showing the autocorrelation coefficient (*r*) between shortest geographic distance by water (km) calculated with *maremap* and genetic differentiation with significance assessed using 1000 permutations. Positive *r* values indicate greater genetic similarity among individuals within a distance class than expected by chance. Autocorrelation coefficients are significant when confidence intervals do not cross the zero‐axis.

Overall, 169 individuals (30.1%) were correctly self‐assigned to the samples from which they originated (excluding samples with *N* ≤ 6). The percentage of successful self‐assignment ranged from 7% (TR_Grimsholmen, *N* = 15 genotyped individuals) to 59% (Porsanger, *N* = 51) (Figure S8). The assignment of the individuals to their counties of origin (once excluded North Trøndelag and Agder due to *N* ≤ 6) revealed that 322 seals (57.3%) were correctly assigned, ranging from 44.2% (Troms) to 81% (Rogaland). The miss‐assignment among the southernmost counties, Telemark, Vestfold, Østfold reflected the lack of genetic differentiation between them (Table 2). Likewise, the low levels of differentiation between Rogaland and Finnmark accounted for the 9.5%–11.4% bidirectional miss‐assignment (i.e., 9.5% of the individuals from Rogaland being assigned to Finnmark, and 11.4% of the individuals of Finnmark being assigned to Rogaland).

**TABLE 2:** GeneClass direct assignment to county for set of 562 individuals once discarded the two samples with *N* ≤ 6: Percentage of individuals coming from the counties indicated in the rows that were assigned to each of the counties indicated in the columns.

	Finnmark	Troms	Nordland	Trøndelag	Møre_Romsdal	Vestland	Rogaland	Telemark	Vestfold	Østfold
Finnmark	**56.3**	13.9	0.6	5.1	2.5	1.9	11.4	3.2	4.4	0.6
Troms	14.0	**44.2**	14.0	4.7	8.1	4.7	2.3	3.5	3.5	1.2
Nordland	12.0	6.8	**53.0**	9.4	2.6	3.4	6.0	1.7	0.9	4.3
Trøndelag	2.3	11.6	4.7	**72.1**	0.0	0.0	2.3	4.7	2.3	0.0
Møre_Romsdal	0.0	5.4	2.7	5.4	**64.9**	0.0	2.7	5.4	8.1	5.4
Vestland	5.3	0.0	0.0	5.3	0.0	**78.9**	0.0	0.0	10.5	0.0
Rogaland	9.5	0.0	0.0	4.8	0.0	4.8	**81.0**	0.0	0.0	0.0
Telemark	4.2	4.2	0.0	4.2	4.2	0.0	4.2	**66.7**	8.3	4.2
Vestfold	5.9	2.9	2.9	2.9	0.0	0.0	2.9	14.7	**52.9**	14.7
Østfold	0.0	0.0	4.3	8.7	4.3	8.7	0.0	8.7	13.0	**52.2**

*Note:* Colour gradient starts in white (0% of individuals assigned) and moves towards dark green with increasing numbers. Numbers in the diagonal highlighted in boldface fonts depicts the self‐assignment, i.e., percentage of individuals correctly assigned to their county of origin.

Sensitivity analysis found a normal L‐shaped allele‐frequency distribution in every sample under all tested conditions, whereas the probabilities of heterozygote excess varied with the assumed TPM and variance parameters (Table S5). In sixteen out of the samples, no evidence of bottleneck was detected under any parameter combination. Only STD_Froan displayed significant heterozygote excess in half of the tests. When applying the parameters used by Andersen et al. (2011) for harbour seals from the same geographic area, none of the samples showed significant heterozygote excess, indicating no evidence of recent bottleneck (Table 1). No heterozygote deficiency was detected either. Together, these findings suggest that the analysed samples are consistent with mutation–drift equilibrium.

## 4 Discussion

Harbour seals' evolutionary histories are shaped by a combination of postglacial recolonization, founder effects, strong philopatry, limited dispersal, genetic drift and local adaptation (Andersen et al. 2011; Blanchet et al. 2021; Liu et al. 2022). Recognising this variation can help identify evolutionary important units for management, something particularly relevant for this species, which faces substantial anthropogenic pressure (e.g., Kovacs and Lydersen 2008; North Atlantic Marine Mammal Commission 2024), and where genetic differences among breeding areas reflect restricted dispersal and long‐term population separation (Olsen et al. 2014).

Population structure of harbour seals has seldom been addressed at local scales, in contrast with large geographic ranges (Andersen and Olsen 2010; Andersen et al. 2011; Burg et al. 1999; Goodman 1998; Olsen et al. 2014, 2017; Westlake and O'Corry‐Crowe 2002) or even global (Liu et al. 2022). Zooming into Norwegian waters, the hypothesis that political borders do not necessarily match biological units gets support in two manners. On the one hand, no genetic differentiation was displayed across the four south‐eastern counties of Agder, Telemark, Vestfold and Østfold. The lack of detectable differentiation suggests that these subpopulations form a relatively connected evolutionary unit, with sufficient gene flow to prevent strong divergence; and therefore, could support a joint management. This solution would align with the outcome of harbour seal telemetry experiments in Vestfold and Telemark, which showed migrations between tagging sites and the neighbouring counties of Østfold and Agder (Riaz et al. 2026). By contrast, the finer‐scale structure observed within geographically large counties—as suggested by STRUCTURE and EEMS analyses—may indicate more independent evolutionary trajectories. This suggests that dividing these counties into smaller subunits could provide a more appropriate basis for management. Thus, Finnmark could be divided, at least, into an eastern and western component while bearing in mind that Porsanger also deviates from the eastern cluster. Likewise, telemetry studies of harbour seals in Porsangerfjord showed that the seals mainly stayed inside the fjord (Ramasco et al. 2017). Nordland, another large county, seemingly consisted of three populations showing significant differentiation among them. Telemetry studies confirmed that harbour seals tagged in Stø, the outer coast in north Nordland, stayed relatively close to the tagging area (Ramasco 2008).

### 4.1 Population Structure and Assignment

Tagging studies have revealed that, although harbour seals may undertake long‐distance foraging migrations, the range and duration of the movements are restricted during mating season, indicative of finer population structure than reported by genetic studies (see Olsen et al. 2014 and references therein). Knowledge of mating season behaviour and genetic analysis shows statistically significant fine‐grain genetic differentiation as harbour seals tend to move within a range of 50–100 km from their natal terrestrial haul out areas (e.g., Andersen and Olsen 2010; Andersen et al. 2011; Goodman 1998; Olsen et al. 2014, 2017; Stanley et al. 1996; Westlake and O'Corry‐Crowe 2002). Furthermore, large open stretches of deep water seemingly constitute barriers to gene flow since harbour seals rarely venture to cross them (Liu et al. 2022 and references therein), which in Norway is reflected by the reduced migration identified by EEMS in the deep and wide Porsangerfjord, in Finnmark (see Figure 7).

Strong natal philopatry resulting in fine‐scale genetic structure and isolation‐by‐distance has been confirmed in the Norwegian coastline using 16 microsatellites. The levels of differentiation detected here matched those reported by Olsen et al. (2014) for southern Scandinavia, thus expanding these authors' finding of fine scale structuring across wider geographic ranges. Patterns of IBD have also been reported for harbour seals in other geographic regions such as across the Atlantic Ocean using mtDNA (Stanley et al. 1996) or in the NE Pacific, where genetic differentiation was detected on a scale of few hundred kilometres along its 16,000 km of continuous distribution (Mizuno et al. 2020; Westlake and O'Corry‐Crowe 2002). In our study, where most of pairwise *F*_ST_ comparisons were statistically significant, shortest waterway distance accounted for 17% of the variation in genetic distance whereas in southern Scandinavia, the pattern of IBD was driven by the differentiation between Skagerrak‐Kattegat‐Western Baltic and Limfjorden‐Wadden Sea region (Olsen et al. 2014). In the Norwegian coastline, in accordance with IBD, individuals from haul‐out sites separated by waterway distances ranging 0–300 km were genetically more similar than expected by chance, which agrees with the species' relatively small‐scale movements described above. However, Rogaland weakens the pattern of IBD due to the unexpected low differentiation towards the geographically distant samples from Finnmark, namely the Porsangerfjord (see Figure 6). If Rogaland was discarded from the IBD analysis, shortest waterway distance would account for 24% of the variation in genetic differentiation. The extended edges connecting Rogaland and Finnmark in the genetic network together with the non‐significant spatial autocorrelation around 1700–1900 km (with *r* around zero) suggest genetic similarity between these geographically distant sites that is not readily explained a simple isolation‐by‐distance model. This pattern could reflect historical gene flow along the Norwegian coast, long‐distance dispersal during non‐breeding periods, or the persistence of shared ancestral variation following postglacial recolonization. If the shared variation included adaptive alleles, it could also have implications for adaptive introgression or evolutionary rescue, although the present data cannot establish this. High‐resolution genomic analyses, combined with tagging and parentage data, could help distinguish among these possibilities. However, values fluctuating around zero after the autocorrelation function crosses zero can arise from several processes, including random variation and therefore they do not, on their own, demonstrate that geographic distance either limits or promotes migration.

The remarkable fluctuations in population size and distribution that harbour seal populations have been experiencing in the North Atlantic are driven by a combination of epizootic diseases such as Phocine Distemper Virus (PDV) with anthropogenic effects such as hunting, by‐catch and habitat destruction (Dietz and Härkönen 1989; Härkönen et al. 2006). In North Norway, i.e., Nordland and Finnmark counties, hunting depleted harbour seals in the beginning of the XXI century (Nilssen et al. 2010), whereas in Skagerrak, the 1988 and 2002 PDV outbreaks caused mass mortalities (Härkönen et al. 2006). The set of microsatellites used in the current study did not detect evidence of bottleneck events in any of the studied samples in agreement with Olsen et al. (2017) when investigating harbour seals in the UK and neighbouring mainland Europe regions (France, the Dutch Wadden Sea and eastern Norway). However, Svalbard, the world's northernmost population and listed on the Norwegian Red List, showed indication of having experienced a bottleneck resulting from a recent population decline (Andersen et al. 2011).

### 4.2 Management Implications

A set of 12 microsatellite loci genetically confirmed the 11 management areas in which Scottish and English harbour seals were formerly distributed based upon tagging data, and distribution of breeding sites and haul‐outs. Samples displaying pairwise *F*_ST_ values ranging between 0 and 0.29 were divided into two major genetic clusters (Olsen et al. 2017).

The coastline of mainland Norway is divided into three zoogeographic sub‐provinces, i.e., Skagerrak (between the Swedish‐Norwegian border and Vest‐Agder), western Norway (from Rogaland northward along the Norwegian west coast including Troms in the north) and Finnmark (north of Troms) (Bjørge et al. 2007). The genetic clustering of harbour seals in Norway—far from being as neat as in the UK—partially aligns with the zoogeographic divisions as three, albeit partially sub‐structured, groups could be identified. The geographic extremes are the ones that match the zoogeographic divisions the best as the southern sub‐province of Skagerrak represents a relatively homogeneous group involving the counties between Agder and Østfold. In the northernmost extreme of Finnmark, Porsanger deviates the most from its cluster, followed by Senja. However, in the western area, the latitudinal gradient of differentiation is interrupted by the very deviating sample from Rogaland as well as a suggestive genetic break in the Nordland samples of NL_Nord_Sto_Had_Rost and NL_Fugl_Nesøy. After distributing the individuals into geographically explicit samples of uneven sizes, most of the pairwise *F*_ST_ values (except in the Skagerrak area) were significantly different from zero such as in the British Isles, although with a lower range of values. Samples from three of the areas in this study, i.e., Rogaland, S. Trøndelag and Troms were genotyped with 13,500 SNPs (Liu et al. 2022) ratifying the levels of differentiation detected here with microsatellites.

In 1997, a management regime delineated upon administrative counties was implemented for harbour seals in Norway. The objective was to secure viable stocks within their natural distribution areas by allocating hunting periods to control population sizes in areas where seal numbers were enough to sustain a harvest (Anon 1990). However, in the light of the genetic data presented here, the current stock delineation may warrant revision to account for the genetic differentiation detected within some of the larger counties, as well as the lack of differentiation observed among others. First, the observed misassignment among counties within the Skagerrak sub‐province likely reflects the null genetic differentiation among them, consistent with telemetry studies (Riaz et al. 2026) and the positive, statistically significant spatial autocorrelation detected at distances of 0–300 km (Figure 8). Together, these findings suggest a high degree of connectivity along the relatively short coastal stretch (approximately 250 km) encompassing the counties of Agder, Telemark, Vestfold and Østfold, thereby supporting the consideration of a joint management approach for this area. Secondly, based on the current set of microsatellite markers, the three northernmost counties—Finnmark, Troms and Nordland—appear to comprise two or more genetically distinct population subunits, a pattern that should be taken into consideration in revised stock delineations. Thus, in Finnmark, significant differences were detected between the eastern and western part of the county, and both areas were significantly differentiated from the semi‐isolated Porsangerfjord. In Troms, the sample from Senja differed significantly from the other samples collected within the county, but not from the adjacent northern part of Nordland County, located less than 400 km away. Samples from the southern part of Nordland County were significantly differentiated both from Northern Nordland and the adjacent county to the south (Northern Trøndelag).

In conclusion, analyses of 16 microsatellite markers genotyped in harbour seals sampled along the entire Norwegian coastline provided valuable new insights into population structure and suggest that current stock delineation may warrant re‐evaluation. However, the resolving power of this marker panel is limited, particularly for detecting weak, recent, or gradual differentiation. Accordingly, while broad patterns may be robust, fine‐scale boundaries, the inferred number of populations, or individual assignments should be interpreted cautiously. Validating these findings with additional independent markers, such as genome‐wide SNPs, would therefore improve confidence. Furthermore, the combination of higher‐resolution genomic markers and tagging experiments could provide a more comprehensive and fine‐scale understanding of population connectivity, structure and differentiation.

## Author Contributions

**María Quintela:** formal analysis (lead), methodology (equal), visualization (lead), writing – original draft (lead), writing – review and editing (equal). **Anne Kirstine Frie:** data curation (supporting), formal analysis (supporting), methodology (equal), writing – review and editing (equal). **Roger Lille‐Langøy:** investigation (lead), methodology (lead), writing – original draft (supporting), writing – review and editing (supporting). **Ralph Tiedemann:** formal analysis (supporting), investigation (supporting), writing – original draft (supporting), writing – review and editing (equal). **Kjell Tormod Nilssen:** conceptualization (lead), data curation (lead), funding acquisition (lead), project administration (lead), writing – original draft (supporting), writing – review and editing (equal).

## Funding

This work was supported by the Norwegian Ministry of Trade, Industry and Fisheries.

## Ethics Statement

Necessary authorisations for sampling DNA from harbour seal pups were obtained from the Norwegian Directorate of Fisheries and from the National animal research authority in the Norwegian Food Safety Authority (Approval FOTS ID 19908; FOTS ID 26962). In protected areas (e.g., nature reserves), permissions for sampling were obtained from the County Governors (Norwegian Environment Agency). Sampling was carried out during calm and good weather conditions, when it was possible to see pups in the water and under the sea surface.

## Conflicts of Interest

This study was supported by the Norwegian Ministry of Trade, Industry and Fisheries. No competing interest exists and there are no financial support or relationships that may pose any kind of conflict.

## Supporting information


**Table S1:** Microsatellite array arranged in multiplex reactions and genotyped on *Phoca vitulina* : Multiplex, locus name, species from which the marker was isolated, primer sequences, type of repeat motif, size ranges and number of alleles both obtained in this study, annealing temperature/number of PCR cycles and bibliographic reference.
**Table S2:** Outlier detection outcome according to Arlequin and BayeScan for the set of 16 microsatellites assessed on the full set of individuals. Boldface type depicts candidate loci to balancing selection according to BayeScan.
**Table S3:** Genetic differentiation between geographically explicit samples of individuals genotyped at 16 microsatellites.
**Table S4:** Popgraphs analyses: Total number and type of connections per sample.
**Table S5:** Bottleneck analyses: Sensitivity analysis assuming single‐step mutations (SMM) in the TPM ranging from 70% to 95% at variances of 12% and 36% respectively. In every case, the mode‐shift test reported normal L‐shaped distribution for all samples. Cells shaded in grey depict samples with low number of individuals (*N* ≤ 6).
**Figure S1:** Detailed maps of sampling locations of harbour seal *Phoca vitulina* along the Norwegian coastline from North to South.
**Figure S2:** Genotype accumulation curve for 573 individuals genotyped at 16 microsatellites after randomly sampling 1000 times the corresponding loci.
**Figure S3:** Puechmaille and Evanno's a posteriori analyses of the outcome of STRUCTURE conducted with and without LOCPRIORS.
**Figure S4:** Barplots of STRUCTURE conducted without any prior information at all the solutions suggested by Puechmaille and Evanno's methods.
**Figure S5:** Barplots of STRUCTURE conducted using LOCPRIORS to assist the clustering at different solutions including those suggested by Puechmaille and Evanno's methods.
**Figure S6:** Isolation by graph distance, IBGD. Spearman's rank correlation coefficient, rho = 0.218, *p* = 0.0015. Geographic distance has been calculated using the R package *marmap* as the shortest distance by water avoiding land masses.
**Figure S7:** Areas where the posterior probability *p*{log(m) > 0|D} exceeds 90% are highlighted in blue, whereas areas where the posterior probability *p*{log(m) < 0|D} exceeds 90% are highlighted in orange. This plot reflects that the areas where the effective migration rates are significantly higher/lower than the overall average rate, i.e., ‘corridors’ and ‘barriers’ and barriers, respectively are neglectable for harbour porpoise in the Norwegian coastline according to the information provided by 16 microsatellite markers.
**Figure S8:** GeneClass analyses: Percentage of individuals correctly assigned per sample. The upper plot represents the samples arranged into latitudinal order along the coastline from the North (Russian border) to the South (Swedish border). The lower plot represents the samples arranged into sampling size: the leftmost ones with *N* = 14–20, the central ones with *N* = 21–39 and the rightmost ones with *N* = 34–58.

## Data Availability

The genotype raw data used in this study can be publicly accessed from The Norwegian Research Information Repository at https://hdl.handle.net/11250/5483815.
