# Supplementary material for: Genetic Structure of Harbour Seals Phoca vitulina vitulina Along the Coast of Norwegian Mainland: Implications for Management
Source: Ecol Evol. 2026 Nov 7;16(11):e74648. doi: 10.1002/ece3.74648 (PMC13743989; doi:10.1002/ece3.74648)
Supplement: Supplementary file 1 [file ECE3-16-e74648-s001.zip › ece374648-sup-0001-Supinfo.docx]

**SUPPLEMENT**

**Genetic structure of Atlantic harbour seal, *Phoca vitulina vitulina* along the Norwegian coastline**

María Quintela^1🖂^, Anne Kirstine Frie^2^, Roger Lille-Langøy^1^, Ralph Tiedemann^3^, Kjell Tormod Nilssen^2^

^1^Institute of Marine Research, Population Genetics, Bergen, Norway, ^2^Institute of Marine Research, Marine Mammals, Tromsø, Norway, ^3^University of Potsdam, Evolutionary Biology & Systematic Zoology, Potsdam, Germany

**Table S1.-** Microsatellite array arranged in multiplex reactions and genotyped on *Phoca vitulina*: Multiplex, locus name, species from which the marker was isolated, primer sequences, type of repeat motif, size ranges and number of alleles both obtained in this study, annealing temperature/number of PCR cycles, and bibliographic reference.

| **Multiplex** | **Locus** | **Origin** | **Primer sequence (5’–3’)** | **Motif** | **Size (bp)** | **No alleles** | **Temp/Cycles** | **Reference** |
| --- | --- | --- | --- | --- | --- | --- | --- | --- |
| 1 | SGPV3 | Harbour seal (*Phoca vitulina*) | F: **PET-**ACATCAACATTCTCAGTATGGGTGG  R: GCAGACAACACCAAGAATGAACCC | Dimer | 245–291 | Discarded | 58 °C / 30X | Goodman (1995) |
| 1 | Hg8.10 | Grey seal (*Halichoerus grypus*) | F: **NED-**AATTCTGAAGCAGCCCAAG  R: GAATTCTTTTCTAGCATAGGTTG | Dimer | 174–186 | 5 | 58 °C / 30X | Allen *et al.* (1995) |
| 1 | Lc26 | Crabeater seal (*Lobodon carcinophagus*) | F: **VIC-**CTCAAGGGACTGAGCCACTCA  R: ACGGCAGGATTCTGAAACACT | Dimer | 304–316 | 5 | 58 °C / 30X | Davis *et al*. (2002) |
| 1 | Lw20 | Weddell seal (*Leptonychotes weddellii*) | F: **6-FAM-**GACTCTTGCCCCCTTCAG  R: GTTTCACAGACCTGCCTCTAGTG | Dimer | 129–137 | 5 | 58 °C / 30X | Davis *et al*. (2002) |
| 1 | TBPV2 | Harbour seal (*Phoca vitulina*) | F: **6-FAM-**CTCTCCCATCCTCATATTA  R: GTACTACCCAATATAGAGAC | Dimer | 249–265 | 9 | 58 °C / 30X | Burg *et al.* (1999) |
| 2 | Hg6.3 | Grey seal (*Halichoerus grypus*) | F: **VIC-**CAGGGGACCTGAGTGCTTATG  R: GACCCAGCATCAGAACTCAAG | Dimer | 228–242 | 8 | 55 °C / 30–32X | Allen *et al.* (1995) |
| 2 | Hgdii | Grey seal (*Halichoerus grypus*) | F: **PET-**ACCTGCCATAGTGCTCATC  R: GAGCCAACTAAGACAAGCC | Dimer | 132–144 | 6 | 55 °C / 30–32X | Allen *et al.* (1995) |
| 2 | Lc18 | Crabeater seal (*Lobodon carcinophagus*) | F: **NED-**ATTCTCCTCTCACCCCTG  R: AATCGGCTGCTGGTAAAT | Dimer | 285–290 | 4 | 55 °C / 30–32X | Davis *et al*. (2002) |
| 2 | Lw7 | Weddell seal (*Leptonychotes weddellii*) | F: **6-FAM-**TGGGCTTTCTACAGTTC  R: ACATAACTCAAGGGACAA | Dimer | 168–178 | 6 | 55 °C / 30–32X | Davis *et al*. (2002) |
| 2 | M11 | Various pinnipeds incl. *Phoca* | F: **NED-**TGTTTCCCAGTTTTACCA  R: TACATTCACAAGGCTCAA | Dimer | 136–152 | 6 | 55 °C / 30–32X | Gemmell *et al.* (1997) |
| 3 | BG | Elephant seal (*Mirounga* sp) | F: **NED-**AATTAGTATGATGCTGGGCTGTC  R: AATTGGGCATGTGATGTGATGAG | Pentamer | 277–307 | 7 | 58 °C / 30X | Rob Slade in Gemmell *et al.* (1997) |
| 3 | Hg4.2 | Grey seal (*Halichoerus grypus*) | F: **VIC-**AATCGAAATGCTGAGCCTCC  R: TGATTTGACTTCCCTTCCCTG | Dimer | 149–172 | 7 | 58 °C / 30X | Allen *et al.* (1995) |
| 3 | Hg6.1 | Grey seal (*Halichoerus grypus*) | F: **6-FAM-**TGCACCAGAGCCTAAGCAGACTG  R: CCACCAGCCAGTTCACCCAG | Dimer | 139–153 | 7 | 58 °C / 30X | Allen *et al.* (1995) |
| 3 | SGPV11 | Harbour seal (*Phoca vitulina*) | F: **PET-**GTGCTGGTGAATTAGCCCATTATAAG  R: CAGAGTAAGCACCCAAGGAGCAG | Dimer | 154–166 | 5 | 58 °C / 30X | Goodman (1995) |
| 4 | Hg8.9 | Grey seal (*Halichoerus grypus*) | F: **6-FAM-**TGTTAACTATCTGGCACAGAGTAAG  R: TTTCCTATGGGTTCTACTCTC | Dimer | 186–190 | 3 | 58 °C / 30–32X | Allen *et al.* (1995) |
| 4 | Lw11 | Weddell seal (*Leptonychotes weddellii*) | F: **VIC-**CTCTCCCTCTCACCTTCC  R: GGCAAATGAGGTGATGTC | Dimer | 152–182 | 9 | 58 °C / 30–32X | Davis *et al*. (2002) |
| 4 | SGPV10 | Harbour seal (*Phoca vitulina*) | F: **PET-**TTCACTTAGCATAATTCCCTC  R: TCATGAATTGGTATTAGACAAAG | Dimer | 135–137 | 2 | 58 °C / 30–32X | Goodman (1995) |

Allen, P. J., Amos, W., Pomeroy, P. P., and Twiss, S. D. 1995. Microsatellite variation in grey seals (*Halichoerus grypus*) shows evidence of genetic differentiation between two British breeding colonies. Molecular Ecology, 4: 653–662.

Burg, T. M., Trites, A. W., and Smith, M. J. 1999. Mitochondrial and microsatellite DNA analyses of harbour seal population structure in the northeast Pacific Ocean. Canadian Journal of Zoology, 77: 930–943.

Davis, C. S., Gelatt, T. S., Siniff, D., and Strobeck, C. 2002. Dinucleotide microsatellite markers from the Antarctic seals and their use in other Pinnipeds. Molecular Ecology Notes, 2: 203–208.

Gemmell, N. J., Allen, P. J., Goodman, S. J., and Reed, J. Z. 1997. Interspecific microsatellite markers for the study of pinniped populations. Molecular Ecology, 6: 661–666.

Goodman, S. J. 1995. Dinucleotide repeat polymorphisms at seven anonymous microsatellite loci cloned from the European harbour seal (*Phoca vitulina vitulina*) Animal Genetics, 28: 310–311.

**Table S2.** Outlier detection outcome according to Arlequin and BayeScan for the set of 16 microsatellites assessed on the full set of individuals. Boldface type depicts candidate loci to balancing selection according to BayeScan.

|  | **Arlequin** | | | **BayeScan** | | |
| --- | --- | --- | --- | --- | --- | --- |
| **Locus** | ***H*_O_** | ***F*_ST_** | ***F*_ST_ *P*-value** | **log_10(_PO)** | **qval** | ***F*_ST_** |
| BG | 0.6913 | 0.0452 | 0.2865 | **3.6988** | **0.0001** | **0.0532** |
| Hg4_2 | 0.3138 | 0.0495 | 0.2359 | 0.5152 | 0.0430 | 0.0762 |
| Hg6_1 | 0.5186 | 0.0206 | 0.2136 | **1000** | **0.0000** | **0.0313** |
| Hg6_3 | 0.6878 | 0.0334 | 0.4435 | **1000** | **0.0000** | **0.0355** |
| Hg8_10 | 0.5394 | 0.0390 | 0.4296 | 1.0935 | 0.0103 | 0.0611 |
| Hg8_9 | 0.4475 | 0.0302 | 0.4070 | 1.4057 | 0.0044 | 0.0480 |
| Hgdii | 0.2424 | 0.0487 | 0.2660 | 0.7794 | 0.0204 | 0.0651 |
| LC18 | 0.4561 | 0.0489 | 0.2453 | 0.7685 | 0.0294 | 0.0630 |
| LC26 | 0.4312 | 0.0291 | 0.3837 | **3.2215** | **0.0001** | **0.0390** |
| LW11 | 0.5700 | 0.0326 | 0.4229 | **3.6988** | **0.0001** | **0.0472** |
| LW20 | 0.4994 | 0.0163 | 0.1552 | **1000** | **0.0000** | **0.0324** |
| LW7 | 0.7411 | 0.0422 | 0.3466 | **3.9999** | **0.0000** | **0.0435** |
| M11 | 0.3850 | 0.0112 | 0.1041 | **1000** | **0.0000** | **0.0220** |
| SGPV10 | 0.4484 | 0.0563 | 0.1505 | -0.5473 | 0.0890 | 0.1133 |
| SGPV11 | 0.5057 | 0.0312 | 0.4188 | **2.1518** | **0.0011** | **0.0449** |
| TBPV2 | 0.5191 | 0.0488 | 0.2459 | **2.6009** | **0.0004** | **0.0549** |

**Table S3.** Genetic differentiation between geographically explicit samples of individuals genotyped at 16 microsatellites. (***NOTE: In a separate excel file***).

**Table S4.** Popgraphs analyses: Total number and type of connections per sample.

|  |  |  | **Type of edge** | | |
| --- | --- | --- | --- | --- | --- |
| **County** | **Sample** | **Total no. edges** | **Compressed** | **Proportional** | **Extended** |
| Finnmark | EF_Sør-Varanger | 5 | 3 | 2 | 0 |
| Finnmark | EF_Tana | 6 | 2 | 3 | 1 |
| Finnmark | EF_Lebesby | 4 | 3 | 1 | 0 |
| Finnmark | EF_Porsanger | 8 | 4 | 3 | 1 |
| Finnmark | WF_Måsøy_Nordkapp | 7 | 4 | 3 | 0 |
| Finnmark | WF_Hasvik | 3 | 2 | 1 | 0 |
| Troms | TR_Grimsholmen | 6 | 5 | 1 | 0 |
| Troms | TR_Måsvær | 4 | 3 | 1 | 0 |
| Troms | TR_Risøya | 4 | 4 | 0 | 0 |
| Troms | TR_Senja | 5 | 4 | 1 | 0 |
| Nordland | NL_Nord_Sto_Had_Rost | 5 | 4 | 1 | 0 |
| Nordland | NL_Fugl_Nesøy | 5 | 2 | 1 | 2 |
| Nordland | NL_Vega | 4 | 2 | 1 | 1 |
| Trøndelag | STD_Froan | 3 | 2 | 1 | 0 |
| Trøndelag | STD_Tarva | 4 | 3 | 1 | 0 |
| Møre_Romsdal | Møre_Romsdal | 5 | 1 | 2 | 2 |
| Vestland | Vestland | 7 | 3 | 3 | 1 |
| Rogaland | Rogaland | 6 | 1 | 4 | 1 |
| Telemark | Telemark | 2 | 2 | 0 | 0 |
| Vestfold | Vestfold | 4 | 2 | 1 | 1 |
| Østfold | Østfold | 5 | 2 | 1 | 2 |

**Table S5.** Bottleneck analyses: Sensitivity analysis assuming single-step mutations (SMM) in the TPM ranging from 70 % to 95 % at variances of 12 % and 36 % respectively. In every case, the mode-shift test reported normal L-shaped distribution for all samples. Cells shaded in grey depict samples with low number of individuals (N≤6).

|  | **Variance 12 %** | | | | | **Variance 36 %** | | | | |
| --- | --- | --- | --- | --- | --- | --- | --- | --- | --- | --- |
| **Sample** | **SMM 70** | **SMM 80** | **SMM 85** | **SMM 90** | **SMM 95** | **SMM 70** | **SMM 80** | **SMM 85** | **SMM 90** | **SMM 95** |
| EF_Sør-Varanger | 0.264 | 0.353 | 0.470 | 0.570 | 0.702 | 0.202 | 0.316 | 0.353 | 0.450 | 0.684 |
| EF_Tana | 0.202 | 0.316 | 0.391 | 0.510 | *0.018* | 0.161 | 0.281 | 0.372 | 0.450 | 0.550 |
| EF_Lebesby | 0.096 | 0.149 | 0.149 | 0.161 | 0.202 | 0.096 | 0.137 | 0.137 | 0.161 | 0.202 |
| EF_Porsanger | 0.530 | 0.609 | 0.719 | 0.826 | 0.863 | 0.490 | 0.550 | 0.666 | 0.812 | 0.851 |
| WF_Måsøy_Nordkapp | 0.570 | 0.647 | 0.666 | 0.702 | 0.736 | 0.470 | 0.570 | 0.609 | 0.702 | 0.768 |
| WF_Hasvik | 0.126 | 0.126 | 0.137 | 0.232 | 0.298 | 0.096 | 0.126 | 0.149 | 0.188 | 0.264 |
| TR_Grimsholmen | *0.047* | 0.094 | 0.115 | 0.151 | 0.244 | *0.036* | 0.076 | 0.084 | 0.126 | 0.195 |
| TR_Måsvær | 0.736 | 0.783 | 0.863 | 0.912 | 0.942 | 0.666 | 0.736 | 0.812 | 0.884 | 0.928 |
| TR_Risøya | 0.116 | 0.188 | 0.232 | 0.264 | 0.334 | 0.106 | 0.149 | 0.217 | 0.248 | 0.334 |
| TR_Senja | *0.037* | 0.106 | 0.137 | 0.248 | *0.000* | *0.022* | 0.052 | 0.106 | 0.202 | 0.281 |
| NL_Nord_Sto_Had_Rost | 0.334 | 0.470 | 0.570 | 0.894 | 0.912 | 0.137 | 0.353 | 0.430 | 0.719 | 0.912 |
| NL_Fugl_Nesøy | 0.470 | 0.550 | 0.628 | 0.702 | 0.752 | 0.391 | 0.510 | 0.550 | 0.647 | 0.768 |
| NL_Vega | 0.609 | 0.719 | 0.798 | 0.839 | 0.433 | 0.590 | 0.684 | 0.719 | 0.839 | 0.894 |
| NTD_Hortavaer | 0.805 | 0.821 | 0.821 | 0.879 | 0.892 | 0.821 | 0.805 | 0.821 | 0.852 | 0.866 |
| STD_Froan | *0.025* | *0.047* | 0.052 | 0.116 | 0.174 | *0.019* | *0.033* | *0.047* | 0.088 | 0.149 |
| STD_Tarva | 0.202 | 0.298 | 0.372 | 0.391 | 0.490 | 0.174 | 0.248 | 0.316 | 0.372 | 0.490 |
| Møre_Romsdal | 0.065 | 0.137 | 0.161 | 0.248 | 0.353 | *0.033* | 0.072 | 0.137 | 0.174 | 0.316 |
| Vestland | *0.047* | 0.106 | 0.137 | 0.149 | *0.002* | *0.047* | 0.058 | 0.080 | 0.126 | 0.188 |
| Rogaland | 0.104 | 0.138 | 0.165 | 0.211 | 0.064 | 0.104 | 0.104 | 0.138 | 0.180 | 0.211 |
| Agder | 0.700 | 0.719 | 0.719 | 0.805 | 0.679 | 0.619 | 0.719 | 0.738 | 0.756 | 0.719 |
| Kragerø | 0.106 | 0.174 | 0.232 | 0.316 | 0.334 | 0.058 | 0.096 | 0.174 | 0.264 | 0.334 |
| Færder | 0.316 | 0.490 | 0.609 | 0.666 | 0.719 | 0.264 | 0.391 | 0.510 | 0.628 | 0.719 |
| Østfold | 0.264 | 0.298 | 0.372 | 0.510 | *0.013* | 0.174 | 0.298 | 0.334 | 0.450 | 0.609 |

**FIGURES**

| a)   | b)   |
| --- | --- |

| c)   | d)   |
| --- | --- |

| e)   | f)   |
| --- | --- |

**Fig. S1.** Detailed maps of sampling locations of harbour seal *Phoca vitulina* along the Norwegian coastline from North to South.

**Fig. S2.** Genotype accumulation curve for 573 individuals genotyped at 16 microsatellites after randomly sampling 1,000 times the corresponding loci.

**Fig. S3.** Puechmaille and Evanno’s *a posteriori* analyses of the outcome of STRUCTURE conducted with and without LOCPRIORS.

NO PRIORS__K=2

NO PRIORS__K=3

NO PRIORS__K=5

**Fig. S4.** Barplots of STRUCTURE conducted without any prior information at all the solutions suggested by Puechmaille and Evanno’s methods.

LOCPRIORS__K=2

LOCPRIORS__K=3

LOCPRIORS__K=4

LOCPRIORS__K=5

LOCPRIORS__K=6

LOCPRIORS__K=7

**Fig. S5.** Barplots of STRUCTURE conducted using LOCPRIORS to assist the clustering at different solutions including those suggested by Puechmaille and Evanno’s methods.

**Fig. S6.** Isolation by graph distance, IBGD. Spearman's rank correlation coefficient, rho=0.218, P=0.0015. Geographic distance has been calculated using the R package *marmap* as the shortest distance by water avoiding land masses.

**Fig. S7.** Areas where the posterior probability P{log(m)>0|D} exceeds 90% are highlighted in blue, whereas areas where the posterior probability P{log(m)<0|D} exceeds 90% are highlighted in orange. This plot reflects that the areas where the effective migration rates are significantly higher/lower than the overall average rate, *i.e.* “corridors” and “barriers” and barriers, respectively are neglectable for harbour porpoise in the Norwegian coastline according to the information provided by 16 microsatellite markers.

**Fig. S8.** GeneClass analyses: Percentage of individuals correctly assigned per sample. The upper plot represents the samples arranged into latitudinal order along the coastline from the North (Russian border) to the South (Swedish border). The lower plot represents the samples arranged into sampling size: the leftmost ones with N=14–20, the central ones with N=21–39 and the rightmost ones with N=34–58.
